# Febrile Neutropenia in the Context of Clozapine-Induced Agranulocytosis (CIA) and Myelodysplastic Syndrome: Importance of a Multidisciplinary Approach

**DOI:** 10.7759/cureus.98805

**Published:** 2025-12-09

**Authors:** André Filipe Conchinha, Tiago Pack, Afonso Rodrigues, Adriana Lopes, António Santos

**Affiliations:** 1 Internal Medicine, Centro Hospital Universitário de Lisboa Central, Lisbon, PRT; 2 Internal Medicine, Hospital dos Lusíadas, Lisbon, PRT; 3 Internal Medicine, Hospital de Santa Marta, Unidade Local de Saúde de São José, Lisbon, PRT

**Keywords:** clozapine-induced agranulocytosis, febrile neutropenia, leukopenia, myelodysplastic syndrome (mds), myelogram

## Abstract

Febrile neutropenia (FN) constitutes a medical emergency, with significant associated mortality, and should be addressed by a multidisciplinary team. We present a case of FN in the context of clozapine-induced agranulocytosis (CIA) superimposed on myelodysplastic syndrome with excess type 2 blasts (MDS-EB2). The mechanism by which clozapine induces agranulocytosis remains unclear, but it represents an idiosyncratic reaction affecting few patients. In the case described, neutropenia maintained a downward kinetics (nadir 0 neutrophils) even after discontinuation of the antipsychotic and the institution of targeted antibiotic therapy, which prompted a myelogram and bone biopsy, consistent with myelodysplastic syndrome. This overlapping of pathologies has contributed to the severity of the neutropenia and leads to a worse prognosis. The complexity of the case required frequent collaboration with other specialties (hematology and psychiatry).

## Introduction

Neutrophils play a key role in the body's defense against infections, particularly in bacterial and fungal infections. Neutropenia is a decrease in the absolute neutrophil count (<1500/μL), and the risk of infection directly depends on its severity and duration [1-3].

Neutrophil depletion can occur through three pathophysiological mechanisms: decreased bone marrow production (congenital abnormalities; hematologic or infiltrative infectious disease; myelotoxic drugs; or nutritional deficiencies); the transfer of circulating neutrophils to secondary lymphoid tissues or the endothelium, a phenomenon called marginalization (as can occur in hypersplenism or splenomegaly); and immune destruction of circulating neutrophils (pharmacological effect or autoimmune mechanism). Treatment should be directed at the underlying cause, and granulocyte colony-stimulating agents may be necessary [3,4]. Febrile neutropenia (FN) is defined as an oral temperature ≥ 38.3°C, or > 38°C maintained for more than one hour, and an absolute neutrophil count < 500/μL, or < 1000/μL with a predicted decline to < 500/μL within the next 48 hours [5]. FN constitutes a medical emergency, as it is associated with high mortality and morbidity, and should be addressed by a multidisciplinary team. Treatment requires early initiation of antibiotic therapy, ideally after appropriate cultures [3-5]. In this case, we describe iatrogenic drug-induced neutropenia in a patient who concomitantly presented a myelodysplastic syndrome with excess type 2 blasts (MDS-EB2). 

## Case presentation

A 71-year-old female patient lived alone and was independent in activities of daily living. She had a known medical history of hypertension, dyslipidemia, hypothyroidism, and bipolar disorder. Her medications included enalapril 5 mg, atorvastatin 20 mg, levothyroxine 75 mg, and lithium carbonate 800 mg. The clozapine 300 mg was started four months back and increased over the past two months.

She presented to the emergency department (ED) due to anorexia and dysuria for one week. Physical examination revealed a good blood pressure profile, fever (tympanic temperature 38.9°C), disorientation in time and space, and dehydrated skin and mucous membranes, with no other notable changes. Laboratory tests revealed leukopenia (0.46 x 10^9/L), neutropenia (0.03 x 10^9/L), a C-reactive protein (CRP) level of 284 mg/L, and a urine test result. The peripheral blood showed macrocytosis, anisocytosis, and basophilic stippling. Urine and blood cultures were taken, and she was started on empirical antibiotic therapy with piperacillin/tazobactam 4.5 g every six hours, assuming FN (Table 1). 

**Table 1:** Evolution of analytical parameters (leukocytes, neutrophils, hemoglobin, platelets, and C-reactive protein) throughout the days of hospitalization.

Test	1^st^ Day	14^th^ Day	16^th^ Day	25^th^ Day	29^th^ Day	35^th^ Day	Unit	Reference value
Leukocytes	0.46	0.52	0.87	24.2	27.3	9.2	x 10^9/L	04-Nov
Neutrophils	0.03	0	0.05	17.3	18.1	4.71	x 10^9/L	2-8.5
Hemoglobin	12.6	10.8	11.4	11.9	13.7	12.4	g/dL	13-18
Platelets	407	645	593	429	327	381	x 10^9/L	150-400
C-reactive protein (CRP)	282.4	144.8	317	64	87.7	20.9	mg/L	<5

She was admitted for etiological evaluation of the FN, which, given the recent initiation of clozapine, was interpreted as clozapine-associated agranulocytosis. She was observed by the psychiatry team, who discontinued clozapine and started quetiapine. Urine cultures grew *Escherichia coli*, which was sensitive to the established antibiotic therapy (sensitive to amoxicillin/clavulanic acid and ceftriaxone; resistant to amoxicillin). She completed the therapy for a total of 14 days. During this period, the patient remained clinically stable, apyretic, and with decreasing neutropenia (reaching 0 x 10^9/L). Due to persistent leukopenia with increased thrombocytosis (maximum of 680 x 10^9/L, likely reactive to the infectious condition), the case was discussed with the hematology department. Other causes of neutropenia (nutritional deficiencies, vitamin B12, and folate deficiencies) were evaluated and did not reveal any abnormalities. Considering the absence of a definitive diagnosis, on the 12^th^ day, myelography and bone marrow biopsy were performed, which revealed myelodysplastic syndrome with 16% blasts. The patient also underwent a computed tomography (CT) of the chest, abdomen, and pelvis, which showed no abnormalities.

On the 16^th^ day of hospitalization, the patient experienced a new fever spike (39.2°C), without symptoms suggestive of an infectious focus. Empirical antibiotic therapy with meropenem 1g every eight hours was initiated after cultures were collected, which were later negative. In the following days, there was a sustained spontaneous increase in leukocytes and neutrophils (maximum 16.07 x 10^9/L). The case was again discussed with Hematology, which, considering the possibility of progression to acute myeloid leukemia, indicated a new bone biopsy (on the 25^th^ day of hospitalization) and the initiation of hydroxyurea 1 g per day (~15 mg/kg/day). After four days, the bone marrow study confirmed MDS-EB (13%), so the hydroxyurea was discontinued, and a gradual decrease in leukocytosis continued to be observed.

It is important to note that, regarding the psychiatric pathology, the patient fluctuated throughout her hospitalization, with periods of increased prostration alternating with psychomotor agitation, culminating in an episode of escape attempts and paranoid ideation directed at healthcare professionals. In this context, and with the psychiatry team recognizing symptoms related to a manic episode, she required the introduction of multiple neuroleptics (olanzapine, haloperidol, and levomepromazine) and lithium, with progressive adjustments. With the adjustments to the neuroleptic medication, the patient became more stable, although some episodes of disorientation and psychomotor impairment persisted.

**Figure 1:**
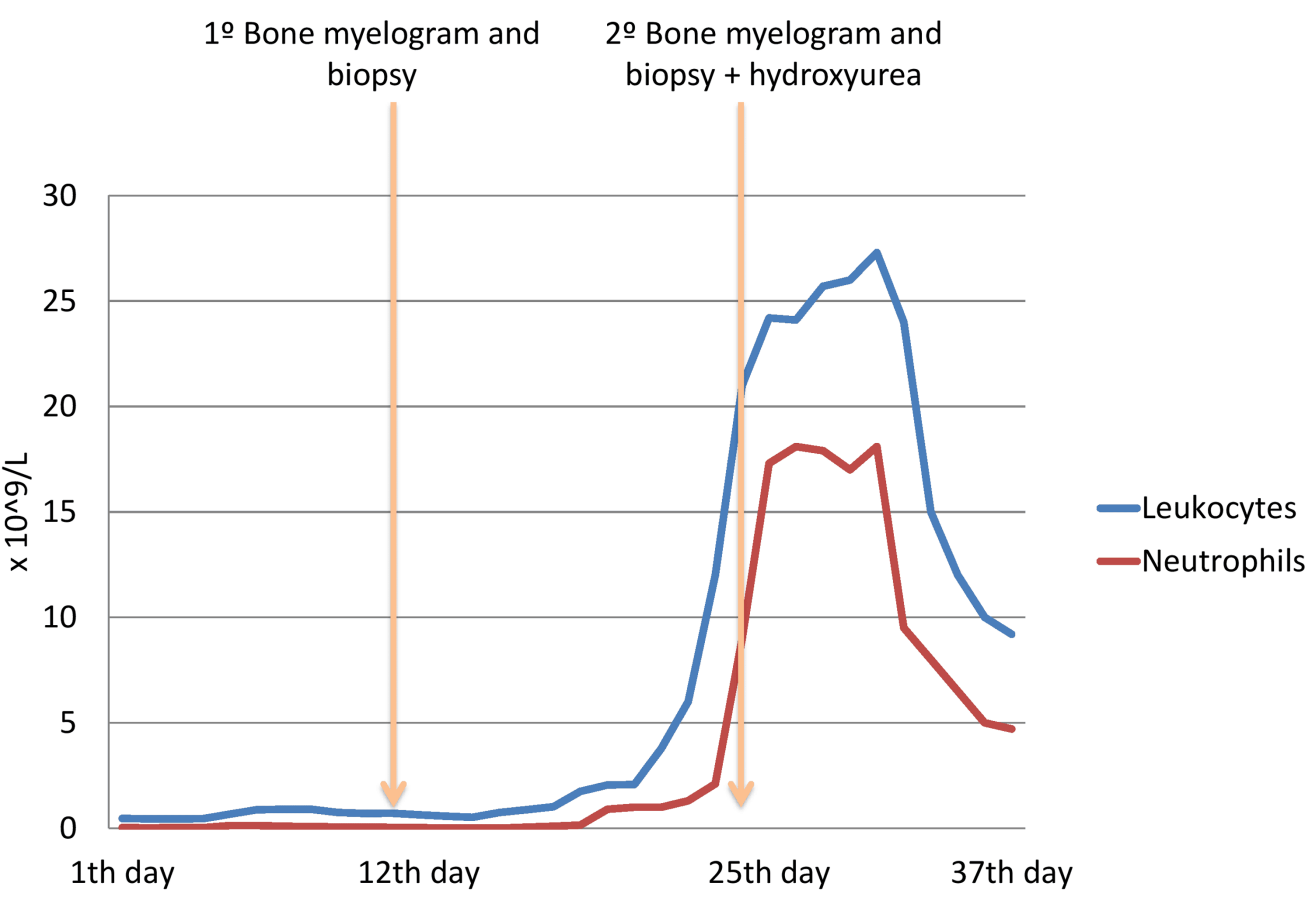
Leukocytes and neutrophils counts evolution over the days of hospitalization.

On the 35^th^ day of hospitalization, the blood count was normalized (hemoglobin 12 g/dl, leukocytes 11.07 X 10⁹/L, neutrophils 4.02 X 10⁹/L, and platelets 172 X 10⁹/L). However, the patient continued to exhibit psychiatric symptoms, including delusional ideas and agitation. In this sense, she was transferred to the Psychiatry Inpatient Service, assuming the diagnosis of clozapine-induced agranulocytosis (CIA) simultaneously with a MDS-EB2 (Figure 1).

After three weeks of hospitalization in the Psychiatry Service, a patient was discharged without psychiatric symptoms, medicated with olanzapine and lithium.

## Discussion

Drug-induced agranulocytosis is a potentially fatal idiosyncratic reaction due to the increased susceptibility to infectious conditions. Among the various causative agents, clozapine is particularly prevalent due to its role in treatment-refractory schizophrenia, for which it is often considered the only effective drug. Clozapine is an atypical antipsychotic, introduced in Europe for the treatment of psychosis in the 1970s, that quickly gained popularity due to its efficacy combined with the absence of extrapyramidal effects. However, the significant risk of neutropenia and agranulocytosis has limited its use to resistant cases, currently requiring regular blood count monitoring [6-8]. 

This entity affects approximately 1% of patients, usually occurring between six and 18 weeks after starting treatment, with an associated mortality rate of 2.7% to 3.1% [6, 7, 9]. The mechanism by which clozapine induces agranulocytosis remains unclear. Some studies have postulated an immune-mediated pathogenesis, while others have sought to identify metabolites toxic to hematopoietic cells, but their results have been inconsistent. However, the research revealed two relevant findings: 1) Statistically, there appears to be a genetic susceptibility in some haplotypes, particularly HLA-B38; 2) CIA is not dose-dependent. Clinically, the manifestations most associated with agranulocytosis are fever and oral ulcers; however, many patients remain completely asymptomatic. Myelograms and bone biopsies are not routinely performed in patients with psychiatric disorders, but they could demonstrate a reduction in granulocytic precursors. If agranulocytosis develops, clozapine should be discontinued immediately. Ideally, the drug should be discontinued before granulocyte counts fall below <500/μL, following the recommended blood count monitoring criteria (the patient in this case missed routine analytical monitoring after starting clozapine). After discontinuation, the average time to recovery is 12 days, which can persist for up to four weeks. Neutropenic patients should be hospitalized, ideally in isolation. The use of granulocyte colony-stimulating agents may be considered to shorten the duration of neutropenia but has not been shown to improve survival in patients with febrile agranulocytosis, which is an emergency and requires early antibiotic therapy [7]. The antibacterial regimen used is determined based on severity, and there are some tools (such as the Multinational Association for Supportive Care in Cancer (MASCC) score) specifically developed for cancer patients that aid in risk assessment [6]. Reintroduction of clozapine may be an option for patients with resistant schizophrenia, particularly if the drug was discontinued due to moderate neutropenia, but it is not advisable if there is a history of prior agranulocytosis [7-10]. In this case, the reintroduction of clozapine is contraindicated, considering the overlap with myelodysplastic syndrome.

In the clinical case described, CIA as the etiology for neutropenia was considered at admission, considering the drug had been administered within the previous 14 weeks. Recovery of blood counts was observed after the median time (17 days after drug discontinuation), which is within the expected recovery based on the literature [7].

In this case, in addition to CIA, a patient presented with MDS-EB2. The diagnostic criteria for MDS-EB2 are as follows: persistent cytopenia in one or more peripheral blood cell lineages (anemia, neutropenia, or thrombocytopenia); morphological dysplasia in one or more bone marrow cell lineages, with ≥10% dysplastic cells; bone marrow blast count of 10% to less than 20% of nucleated cells, or 5% to 19% blasts in peripheral blood; and no evidence of acute myeloid leukemia, which is defined by ≥20% blasts. The superposition of this myelodysplastic syndrome to agranulocytosis could have led to the worsening and persistence of neutropenia. On the other hand, MDS-EB2 presents a high risk of progression to acute myeloid leukemia. Survival in patients with MDS-EB2 is two to three years after diagnosis [11, 12].

This case has been documented as a mutation in the SRSF2 gene, which does not represent a high-risk genetic marker. After discharge from the psychiatry department, the patient continued follow-up in hematology, having started treatment for MDS-EB2 with azacitidine. If her psychiatric condition remains stable, she may be eligible for allogeneic hematopoietic stem cell transplantation in the future, the only curative treatment [12].

Regarding neutropenia, many of the recommendations for treating FN come from oncology guidelines. The American Society of Clinical Oncology (ASCO) and the Infectious Diseases Society of America (IDSA) recommend that empiric antibiotic therapy be initiated within one hour of presentation for any patient with FN. The rationale is that neutropenic patients are at high risk for rapid progression to severe sepsis and death. For initial empiric therapy in most patients, monotherapy with an intravenous antipseudomonal beta-lactam agent (such as cefepime, piperacillin-tazobactam, or a carbapenem like meropenem) is recommended. These recommendations are based on evidence that prompt, broad-spectrum antibiotic coverage reduces morbidity and mortality in FN [1,13]. In this case, antibiotic therapy with piperacillin/tazobactam was also initiated early, in accordance with recommendations.

## Conclusions

FN constitutes a medical emergency, as it is associated with high morbidity and mortality. Prompt initiation of empirical antibiotic therapy, according to the estimated severity, is crucial for prognosis. We describe a case of FN in the context of clozapine agranulocytosis superimposed on MDS-EB2. As indicated in the literature, the patient started early empirical broad-spectrum antibiotic therapy with piperacillin/tazobactam. The overlap of two uncommon etiological factors required ongoing coordination between different specialties.

The authors intend to emphasize that the persistence of iatrogenic neutropenia due to medication led to further etiological investigation, with documentation of a myelodysplastic syndrome. MDS-EB2 has a worse prognosis and requires careful monitoring by hematology.
